# Nickel Ion Release from Three Types of Nickel-titanium-based Orthodontic Archwires in the As-received State and After Oral Simulation

**DOI:** 10.5681/joddd.2014.013

**Published:** 2014-06-11

**Authors:** Barat Ali Ramazanzadeh, Farzaneh Ahrari, Berahman Sabzevari, Samaneh Habibi

**Affiliations:** ^1^Professor, Department of Orthodontics, School of Dentistry, Mashhad University of Medical Sciences, Mashhad, Iran; ^2^Assistant Professor of Orthodontics, Dental Research Center, Mashhad University of Medical Sciences, Mashhad, Iran; ^3^Department of Orthodontics, School of Dentistry, Rafsanjan University of Medical Sciences, Rafsanjan, Iran; ^4^School of Dentistry, Mashhad University of Medical Sciences, Mashhad, Iran

**Keywords:** Copper NiTi, ion release, spectrophotometry, supercable

## Abstract

***Background and aims.*** This study aimed to investigate release of nickel ion from three types of nickel-titanium-based wires in the as-received state and after immersion in a simulated oral environment.

***Materials and methods.*** Forty specimens from each of the single-strand NiTi (Rematitan "Lite"), multi-strand NiTi (SPEED Supercable) and Copper NiTi (Damon Copper NiTi) were selected. Twenty specimens from each type were used in the as-received state and the others were kept in deflected state at 37ºC for 2 months followed by autoclave sterilization. The as-received and recycled wire specimens were immersed in glass bottles containing 1.8 mL of artificial saliva for 28 days and the amount of nickel ion released into the electrolyte was determined using atomic absorption spectrophotometry.

***Results.*** The single-strand NiTi released the highest quantity of nickel ion in the as-received state and the multi-strand NiTi showed the highest ion release after oral simulation. The quantity of nickelion released from Damon Copper NiTi was the lowest in both conditions. Oral simulation followed by sterilization did not have a significant influence on nickel ion release from multi-strand NiTi and Damon Copper NiTi wires, but single-strand NiTi released statistically lower quantities of nickel ion after oral simulation.

*** Conclusion.*** The multi-strand nature of Supercable did not enhance the potential of corrosion after immersion in the simulated oral environment. In vitro use of nickel-titanium-based archwires followed by sterilization did not significantly increase the amount of nickel ion released from these wires.

## Introduction

Biocompatibility of orthodontic materials has long been considered a concern for clinicians due to the long period of orthodontic treatment and the high incidence of allergic reactions to metal appliances.^1^ Nickel, a main ingredient of orthodontic materials, can cause severe health hazards in biologic tissues. Hypersensitivity is the most common consequence of exposure to nickel-containing products, with incidence ranging from 4.5% to 20% in the literature.^-5^ The cytotoxic effect of nickel has been shown in cell culture studies.^6-8^ Subtoxic levels of nickel are capable of causing DNA strand breaks and DNA base damage^9^ and inhibition of DNA lesions repair.^10^ Small quantities of nickel ion are capable of activating monocytes and possibly enhance an inflammatory response in soft tissues.^8^

Because of their excellent mechanical properties of superelasticity, great working range and shape memory, nickel titanium alloys have widespread use in clinical orthodontics, especially in the early stages of treatment for leveling and aligning malposed teeth. These alloys contain nickel and titanium ions in a relatively similar weight ratio. Despite the high content of nickel in NiTi archwires, nickel release from these wires has been demonstrated to be low and under the required threshold to cause biological effects.^6,11-12^, This has been attributed to the spontaneous formation of titanium oxide layer on the surface,^13^ which protects the alloy from corrosion, thus limiting outward ion movement. However, the presence of low pH and high temperature expedite corrosion and nickel ion release from nickel titanium alloys to the levels that are several folds greater than those occurring in higher pH and lower temperature.^11,14-15^ The protective oxide layer on the surface of NiTi wires is also degraded in fluoride-containing environments, leading to corrosion and degradation in mechanical properties of NiTi wires.^16-19^ Under these conditions, the amount of nickel released from nickel titanium alloys may be sufficient to induce allergic reactions at least in patients with a clinical history of hypersensitivity to metal appliances. Previous studies have shown that exposure to nickel-containing orthodontic appliances produce nickel hypersensitivity in subjects sensitized to nickel before initiating therapy.^4,20^

Today, advances in material technology have resulted in the development of superelastic nickel-titanium-based wires with greater range of deflection while exerting dramatically lower force magnitude in the plateau region compared to the conventional NiTi archwires. Supercable is a seven-strand nickel titanium coaxial archwire introduced by Strite Industries and has been advocated for engaging severely crowded teeth and high-buccal canines without undergoing plastic deformation. Damon Copper NiTi was introduced by Ormco for initial tooth alignment, especially when using Damon self–ligating brackets in order to give optimal force range and increase the rate of tooth movement. The superior load-defection properties of Supercable and Damon Copper NiTi archwires have been demonstrated in previous studies,^19,21^ but no study has evaluated the release of nickel ion from these orthodontic archwires in the as-received state and after exposure to a simulated oral environment. The multi-strand configuration in Supercable lowers the stiffness, but it may make the wire susceptible to corrosion, which in turn would affect the quantity of nickel ion release.

The NiTi wires may remain in the oral cavity for several months in strained conditions, and this might affect the corrosion resistance of the alloy. Due to the relatively high cost of Supercable and Damon Copper NiTi, the release of nickel ion from these wires after simulated clinical use and sterilization is also worthy of investigation. The aim of this study was to determine and compare the quantity of nickel ion released from single-strand Ni-Ti, multi-strand Ni-Ti and Damon Copper Ni-Ti wires in the as-received condition and after storage in a simulated oral environment followed by autoclave sterilization.

## Materials and Methods

Three types of nickel-titanium-based archwires, including Rematitan “Lite” (Dentaurum, Ispringen, Germany), Damon Copper NiTi (Ormco Corp., Glendora, CA, USA) and SPEED Supercable (Strite Industries, Cambridge, Ontario, Canada), were selected. The first two archwires are single-strand, whereas the third is a multi-strand wire (Table 1). Forty specimens from each type were obtained by cutting 2-cm lengths from the straight posterior parts of maxillary 0.016-inch round NiTi wires. For each wire, twenty specimens were used in the “as-received” state and the others were kept for 2 months in a simulated oral environment (groups 1 to 6, Table 1). Overall, sixty preformed archwires were used to provide 120 wire specimens.

**Table 1:** Nickel-titanium-based wires used in this study classified by commercial name, composition, number of strands and test condition

Group	Number	Commercial name	Composition	Number of strands	Test condition
1	20	Rematitan “Lite”	NiTi	Single-strand	As-received
2	20				Recycled
3	20	Damon Copper NiTi	Copper NiTi	Single-strand	As-received
4	20				Recycled
5	20	Supercable	NiTi	Multi-strand	As-received
6	20				Recycled

**Figure 1.**
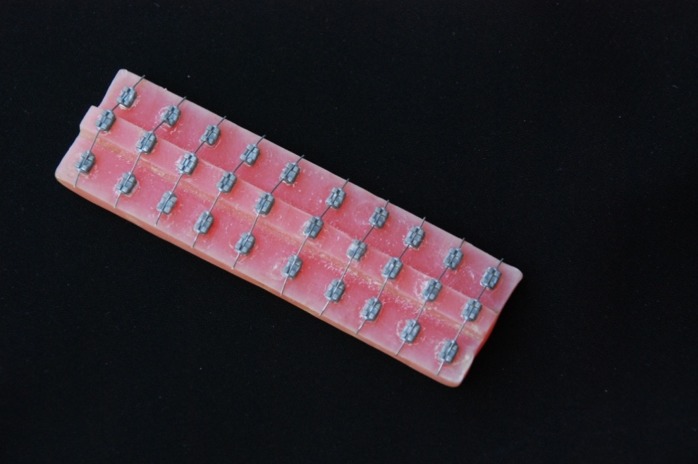
An acrylic model for holding wire specimens in deflected state over a 2-month period.

For oral simulation, acrylic plates were used on each 3-mm step designed to represent moderate crowding in the anterior segment of the maxillary dentition. Ten rows of brackets were bonded to each acrylic model using self-curing acrylic resin.Figure 1 Each row contained three standard edgewise upper central incisor brackets of 0.018-inch slot size (Dentaurum, Ispringen, Germany), which were positioned at the same line with their long axis parallel. The wire specimens were held on the brackets by using elastomeric ligatures (Ortho Technology, Tampa, Florida, USA). The acrylic models were placed in plastic containers filled with Fusayama Meyer artificial saliva solution and kept in a 37°C incubator for 2 months. The artificial saliva with a pH value of 7.03 consisted of KCl (0.4 g/L), NaCl (0.4 g/L), CaCl_2_.2H_2_O (0.906 g/L), NaH_2_PO_4_.2H_2_O (0.690 g/L), Na_2_S.9H_2_O (0.005 g/L) and Urea (1 g/L).^22^ At the end of the 2-month incubation, the specimens were retrieved from the solution, washed and dried. The wires were then subjected to a thermocycling process for 500 cycles, using 5° and 55°C temperatures with a dwell time of 30 seconds per bath. Finally, the specimens were sterilized in an autoclave at 121°C (250°F) and 15 to 20 psi for 20 minutes.

### Ion Release Determination 

For ion release analysis, the specimens in groups 1, 2, 3, 4, 5 and 6 were placed in individual glass bottles containing 1.8 mL of Fusayama Meyer artificial saliva solution. The bottles were kept in an incubator at 37ºC for 28 days and then the nickel ion concentration in the electrolyte was detected using a graphite-furnace atomic absorption spectrophotometer (Perkin Elmer, Model 4100 ZL, Norwalk, CT, USA).

###  Statistical Analysis

One-sample Kolmogorov-Smirnov test showed that the data distribution was normal; therefore, two-way ANOVA was used to compare nickel release from the as-received and recycled specimens of different NiTi wires. The analysis was performed with SPSS 16.0 (SPSS Inc, Chicago, Ill, USA), and the significance level was predetermined at P < 0.05.

## Results

Table 2 displays the concentrations of nickel ions released into the electrolyte for the three types of wires in the as-received state and after oral simulation. Statistical analysis exhibited a significant interaction between the wire type and wire treatment (P<0.001), making it necessary to evaluate these variables separately. Therefore, one-way ANOVA was run, which delineated statistically significant differences between the release of nickel ion from the three types of wires in the as-received state (P<0.001), and after oral simulation (P<0.001) (Table 2). Pairwise comparisons by Tukey tests revealed that among the as-received wires, Rematitan “Lite” released significantly greater quantities of nickel ion compared to Supercable (P<0.05), and Supercable released significantly greater amounts of nickel ion compared to Damon Copper NiTi wire (P<0.05). After oral simulation, the highest and the lowest nickel concentrations in the electrolyte were observed with Supercable and Damon copper NiTi wires, respectively (P<0.05) (Table 2).

**Table 2:** Descriptive statistics and comparison of nickel ion concentration in the electrolyte (µg/L) between the three wires in the as-received state and after oral simulation

Type of wire	As-received state			After oral simulation			P-value (t-test)
	Mean	SD	Pairwise Comparisons^**^	Mean	SD	Pairwise Comparisons^**^	
Rematitan “Lite”	53.96	16.81	a	21.7	11.90	b	< 0.001^*^
Damon Copper NiTi	5.7	1.42	c	7.17	3.60	c	0.104
Supercable	40.3	21.18	b	47.23	17.09	a	0.262
P-value (ANOVA)	< 0.001^*^			< 0.001^*^			
^*^ Statistically significant difference at p < 0.05							
^**^ Different letters indicate statistically significant differences at p < 0.05							

**Figure 2.**
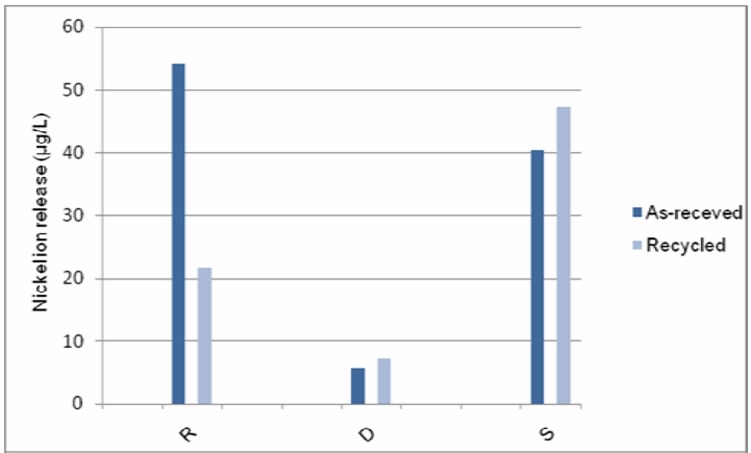
Comparison of nickel ion release (μg/L) from different nickel-titanium-based wires in the as-received state and after oral simulation.

Student’s t-test revealed that the nickel ion concentrations in the electrolyte were not statistically different between the as-received state and after oral simulation in Supercable and Damon Copper NiTi groups, whereas Rematitan “Lite” released significantly lower quantities of nickel after immersion in the simulated oral environment (Table 2). Figure 2 compares the quantities of nickel ion released from the tested wires in the as-received state and after oral simulation.

## Discussion

The present investigation evaluated the quantity of nickel ion released from different nickel-titanium-based wires in the as-received state and after oral simulation. In order to replicate the clinical conditions, acrylic models were used to keep wires in the crowded space during a 2-month interval and the specimens were subjected to thermocycling and sterilization processes. Jia et al^6^ demonstrated that cycling straining caused a significant increase in nickel ion release from NiTi wires immersed in an artificial saliva solution. Another option used in some studies is to determine the nickel ion release of retrieved wires, which had been engaged in the oral environment for 1 month or more.^10,23^ The nickel ion content was measured over 28 days because a previous study found that the metal ion release from orthodontic appliances is almost complete within 4 weeks.^24^

Among the tested wires, Rematitan “Lite” released the highest amount of nickel in the as-received state and Supercable exhibited the highest release after oral simulation. In both conditions, the lowest quantity of nickel ion in the electrolyte was found in Damon Copper NiTi group, which might be attributed to the addition of a small amount of copper into the alloy structure, which has been shown to lower the reactivity of titanium, thus increasing corrosion resistance and biocompatibility of copper-containing NiTi wires.^14^ The findings of this study indicated that in nickel-sensitive patients, use of Copper NiTi wires should be preferred to nickel-titanium archwires.

The results of this study showed no significant difference in nickel ion release from the as-received and recycled Damon Copper NiTi specimens. Despite its multi-strand nature, which may increase the possibility of corrosion, Supercable did not show a significant increase in nickel concentration in the electrolyte after immersion, either. On the other hand, oral simulation had a significant influence on the quantity of nickel ion released from single-strand NiTi wire, in such a way that Rematitan “Lite” released lower amounts of nickel after oral simulation compared to the as-received condition. Poosti et al^23^ also found a smaller amount of nickel ion release after clinical use and wet sterilization of a superelastic nickel titanium wire (Rematitan “Lite”) compared to the as-received specimens, but the difference was negligible and not statistically significant.

Several studies have shown that the nickel-titanium archwires release greater amounts of nickel ion into the electrolyte in the first day or week of immersion and the release is significantly slowed down during the following immersion periods.^11,15,25-26^, This has been attributed to the growing layer of titanium oxide on the surface of NiTi wires, which prevents further diffusion of the alloy constituents into the aqueous environment.^11,15^ Therefore, it can be assumed that the nickel ion release from Rematitan “Lite” mainly occurs over the oral simulation period; therefore, when the wires were immersed in the electrolyte for ion detection analysis, the amount of release significantly decreased compared to that of the as-received specimens. Although this study did not evaluate the net effect of sterilization on nickel ion release from recycled archwires, previous authors have demonstrated that sterilization of NiTi wires by dry heat or steam autoclave had no significant influence on surface parameters or the amount of nickel ion released from NiTi wires.^23,27^

The nickel ion release from new and used nickel-titanium archwires has been investigated in previous studies. It is believed that the release of metal ions from the alloys is enhanced by surface corrosion.^10,14^ Several studies have found surface defects, pitting and crevice corrosion on the surface of NiTi wires immersed in the electrolyte or exposed to the oral environment.^11,28-29^, However, Grimsdottir and Hensten-Pettersen^30^ found no detectable difference in surface topography or composition between used and as-received NiTi archwires. Gursoy et al^24^ found that despite the presence of pitting corrosion on the surface of recycled NiTi wires, the recycling process did not result in significantly greater amount of metal ion release from NiTi archwires. Eliades et al^10^ indicated that nickel content of as-received and retrieved NiTi archwires were similar, implying that no release took place in vivo.^10^

The average range of daily dietary intake of nickel is about 200-300 µg,^15,31-32^, but eating some foods like legumes, spinach, lettuce, grains, baking powder, and cocoa can provide excessive amounts of nickel for the body.^31^ In the present study, the mean quantities of nickel ion released from the 2-cm length archwires over a 28-day interval ranged from 5.7 µg/L (Damon Copper NiTi) to 54 µg/L (Rematitan “Lite”) for the as-received specimens. For recycled wires, the highest and the lowest quantities of accumulated nickel ion were 7.2 µg/L (Damon Copper NiTi) and 47.2 µg/L (Supercable) after 28 days, respectively. In a full-mouth appliance, including upper and lower jaws, the overall length of NiTi archwires exposed to the saliva is around 28 cm and thus the cumulative nickel release from the two as-received nickel-titanium-based archwires would be in the range of 79.8-756 µg/L. The corresponding value for recycled wires is estimated to be 100.8-660.8 µg/L. If we assume that nickel ion release occurs evenly during the 28-day interval, the daily amount of nickel ion released from new and recycled wires would be modest in comparison with the amounts ingested during daily food intake, which is well under the critical threshold of 600‒2500 µg/L over a 24-hour period required to cause allergic reactions.^23^ However, if these levels are added to the nickel ions from dietary sources and that released from other parts of fixed orthodontic appliances, such as bands and brackets, the nickel ion concentration may come close to the critical level required for induction of nickel hypersensitivity. Bass et al^33^ noticed the risk of sensitizing patients to nickel following long-term exposure to nickel-containing orthodontic appliances. Furthermore, in the clinical conditions, the wires are subjected to loads of mastication and tooth brushing and the temperature and chemical conditions are variable. These may contribute to degradation of the protective oxide layer on the surface of nickel titanium archwires, leading to more ion release than that of in vitro conditions. Other predisposing factors such as oral bacterial flora, salivary enzymes, ingested fluids, physical and chemical properties of foods and liquids, acidic environment, and flow rate of saliva can also accelerate metal corrosion and subsequent ion release from orthodontic archwires in the oral environment.^32,34^ It is difficult to encompass all predisposing factors in experimental conditions; therefore, the results of this study should be interpreted within the limitations of in vitro studies. Further studies should be designed to measure the nickel content in oral tissues and its possible adverse cellular interactions.

## Conclusion

1. In the as-received condition, the single-strand NiTi wire (Rematitan “Lite”) produced the highest concentration of nickel in the electrolyte. After oral simulation, the greatest quantity of nickel pertained to the multi-strand NiTi wire (Supercable).
2. Damon Copper NiTi showed the lowest amount of nickel in the electrolyte in both the as-received state and after oral simulation, which may be due to the presence of copper in the alloy structure.
3. The prolonged use of the tested nickel-titanium-based wires followed by sterilization would not increase the risk of corrosion under the conditions used in this study.
